# Medico-Chirurgical Transactions, Published by the Royal Medical and Chirurgical Society of London. Second Series, Volume the Eighth

**Published:** 1844-01-01

**Authors:** 


MEDICO-CHIRURGICAL TRANSACTIONS, PUBLISHED BY THE ROYAL MEDICAL AND CHIRURGICAL SOCIETY OF LONDON. Second Series, Volume the Eighth. 8vo. pp. 425. London, 1843.

THE volume before us is characterised rather by the number than the length of the Papers it contains. Hints, remarks, observations, cases, take the place of essays, and of bulky contributions. This is to be expected. A volume which appears twice in the twelve months, must be so filled, or not at all.

The bill of fare on this occasion is a long one. We have—

1. Case of paralysis without loss of sensation, from disease of the cervical medulla; by John Webster, M.D. 2. Case of bronchial calculus, with observations on disease of the bronchial glands; by John Charles Graham Tice, M.D. 3. On congestive pneumonia consequent upon surgical operations, diseases, and injuries; by John Erichsen, Esq. 4. Researches into the connexion existing between an unnatural degree of compression of the blood contained in the renal vessels, and the presence of certain abnormal matters in the urine; by George Robinson, Esq. 5. An account of an unusually large biliary calculus voided from the rectum; by James Arthur Wilson, M.D. 6. On fatty degeneration of the arteries, with a note on some other fatty degenerations; by George Gulliver, Esq. F.R.S. 7. Remarks on the calculi in St. George's Hospital; by Henry Bence Jones, M.A. 8. Cases of ulceration of the internal jugular vein communicating with an abscess; by W. Bloxam, Esq. 9. Some account of an hysterical affection of the vocal apparatus, with several cases; by Oscar Clayton, Esq. 10. Case of erectile tumor in the popliteal space,—Removal; by Robert Liston, Esq. F.R.S. 11. Two cases of osteo-sarcoma of the thigh bone, requiring amputation of the limb in both instances; by S. A. Frogley, Esq. 12. Remarks on cancrum oris and the gangrenous erosion of the cheek of Mr. Dease and Dr. Underwood, and more particularly on the efficacy of the chlorate of potash in the treatment of those diseases; by Henry Hunt, M.D. 13. Case of ulceration of the pulmonary artery into an abscess of the lungs. With remarks by John Dalrymple, Esq.; by William Crowfoot, jun. Esq. 14. Cases of strangulated hernia reduced "*en masse*," with observations; by James Luke, Esq. 15. Observations on the medicinal properties of the Cannabis Sativa of India; by John Clendinning, M.D. F.R.S. 16. On the sugar in diabetic blood; by Henry Bence Jones, M.A. 17. A few observations on encysted hydrocele; by Robert Liston, F.R.S. 18. Some account of an epidemic which prevailed at Teheran, in the months of January and February, 1843; by C. W. Bell, M.D. 19. On the nature of the ossification of encysted tumors; by John Dalrymple, Esq, 20. On the anatomical characters of some adventitious structures, being an attempt to point out the relation between the microscopic characters and those which are discernible by the naked eye; by Thomas Hodgkin, M.D. 21. An account of a case in which a foreign body was lodged in the right bronchus; by Sir B. C. Brodie, Bart. F.R.S. 22. Second series of observations on the pathology of the ear, based on one hundred and twenty dissections of that organ; by Joseph Toynbee, F.R.S. 23. On the effects of rickets on the growth of the skull; by Alexander Shaw, Esq. 24. On the presence of spermatozoa in common hydrocele; by E. A. Lloyd, Esq. 25. Statistics of Bethlem Hospital; with remarks on insanity; by John Webster, M.D.

The first case to which we shall advert is—

### I. CASE OF PARALYSIS, WITHOUT LOSS OF SENSATION, FROM DISEASE OF THE CERVICAL MEDULLA. By *John Webster*, M.D,

The patient was a gentleman, aged 36, of a muscular frame, who had enjoyed good health, with the exception of headaches, until a few years before his death. In 1836, he suffered from a phagedænic ulcer on the left leg. It broke out again in 1838, and was followed by a large chronic ulcer on the posterior part of the pharynx. The patient had never had any syphilitic affection. His health now suffered, and a severe domestic affliction contributed still further to depress it.

In the Autumn of 1839, Mr. G. began to suffer from almost constant pains in the head, occasionally very severe, and accompanied by sickness and prostration of strength. In January, 1840, slight epileptic attacks supervened, with a pulse as low as 35 or 40. In March, the epileptic attacks were more pronounced and were followed by violent spasmodic contractions of the lower extremities. In talking, Mr. G. often repeated ssveral times the principal word in a sentence, or used a different one from what he wished to employ, being aware, the while, of the error.

In the Summer of 1840, Mr. G. complained of much weakness in the back and loins, with pain in the head and nape of the neck. He soon afterwards became unable to walk steadily without support; and, to use the patient's own words, 'he felt as if his body were cut in two, and the lower half falling away from the upper.' Both hands and arms now became very weak, and were soon nearly powerless; and he also complained of considerable pain about the fourth cervical vertebra, increased in severity on merely bending his head backwards; but this sensation felt however less painful when rotatory motion of the neck was only attempted. In the Autumn, he improved so much as to be able to walk with a staff, and even visit his friends. But after dining out on Christmas-day, and ineffectually attempting to walk home through the snow, the symptoms returned in an aggravated form. He entirely lost the use of the legs and arms, then of the muscles of the abdomen and chest, the respiration being solely carried on by the diaphragm. Very active purgatives and strong enemata were required for the bowels, the frequent employment of the catheter for the bladder. The sense of touch was even exaggerated over the body, a draught of cold air being felt with intensity. Frequently, Mr. G. complained of intense heat of the surface, when to a by-stander, it seemed cold, and at other times, he had alternations of heat and cold, his skin appearing, the while, to remain of the natural temperature. If a foot or toe were touched, he was aware of the exact spot, and spasmodic twitchings of the limb, with much pain, were occasioned. These twitchings were latterly so violent, as almost to throw the patient off his couch.

Towards the last, the urine deposited a thick ropy sediment, and was passed in drops involuntarily; offensive, watery fæces being voided in a similar manner. On the 22nd of July, 1842, the patient died exhausted, retaining his intellectual faculties to the last.

All treatment was ineffectual, and the patient having been under the hands of Dr. Chambers, Dr. Seymour, Dr. Webster, Sir B. Brodie, and Mr. Tatum, is a sufficient guarantee for every remedy likely to prove serviceable having been employed. Morphia, latterly, gave much relief.

*Autopsy.*—Body emaciated. Some effusion of lymph was found under the arachnoid membrane covering the left side of the brain, along with turgescence of that and the other hemisphere; both divisions being pale, and exhibiting a watery aspect, although their texture was firm and compacted. The ventricles of the brain seemed large, particularly the left; and about two ounces of serum were effused in these cavities; the foramen commune being at the same time larger than natural. The arachnoid tissue extending over the pons Varolii adhered to the parietal layer of that membrane; but no tumor, or any other change of structure, was found either in the brain or cerebellum, excepting that the latter organ appeared anæmic, and rather softer in texture than ordinary.

Nothing worth noting in the thorax. The kidneys were somewhat enlarged, anæmic, and exhibited marks of chronic inflammation on the internal membrane of the pelvis and infundibula. The omentum and some of the small intestines adhered to the bladder, the muscular coat of which was greatly thickened, and its cavity so contracted as not to exceed that of an ordinary-sized walnut.

"Having carefully laid open the vertebral column, throughout its whole length, the theca, corresponding to the three or four lower cervical vertebræ, was found to be much distended; and on being cut into, the arachnoid cavity, with the sub-arachnoid tissue, appeared filled with lymph, which evidently had been some time effused: as the membranes were thereby united to each other, and also to the cord. On making a more minute examination of the parts, the adhesions of the membranes to the cord were discovered to be much firmer at its anterior, than posterior portion; indeed they were actually so strong, as to be inseparable from the medulla without rupture. At this particular part, the medulla also appeared larger than usual, felt soft and pulpy to the touch, and on being divided by the knife, its substance seemed to be in an almost diffluent state, infiltrated with serum, but exhibiting a natural colour. For the extent of half an inch above the point just described, the cord had a dusky red tinge, appearing, however, of the ordinary consistence. In the anterior and posterior columns, not much difference was observable to the naked eye, at the first superficial examination of the diseased part of the medulla; although both divisions of the cord seemed considerably softened, infiltrated and disorganised, particularly in the posterior columns; whilst as well above, as below the affected portion, the medulla was healthy, and quite natural in appearance." 14.

A portion of the cord was submitted to Dr. Todd, of King's College, for the purpose of microscopical examination. The following is his Report:—

"'The portion of spinal cord submitted to me by Dr. Webster, appears to consist of the greater part of the cervical segment. I find great destruction (from softening) of the medullary substance of the posterior columns, especially that of the right side; the antero-lateral columns seem to have been also the seat of the softening process to a less degree, but I do not find that they have suffered any loss of substance. In examining the softened parts by the microscope, I detected very few of the proper nerve-tubes; and those which I did observe, were much altered from their natural appearance; they had become opaque, and had assumed an indistinctly fibrous aspect. I was unable to find any trace of grey matter. The posterior horns must have shared in the great destruction of the posterior columns, and probably the anterior ones experienced a similar fate. It is right, however, to observe, that the specimen had been preserved some time in spirits, before it came into my hands. I found throughout the diseased part, numerous small scales, (cholesterine?) such as are very commonly met with in portions of the nervous centres preserved in spirits.'

"Dr. Todd then makes the subsequent very important remarks:—'I consider this case of the greatest physiological interest, as affording unequivocal proof, that the posterior columns cannot perform the office assigned to them by some physiologists, namely, that of conducting sensitive impressions to the brain, or at least, that they are not the only channels of this communication. It is also important in an anatomical point of view, as showing that the posterior roots of the nerves are independent of the posterior columns of the spinal cord; for, although the latter were destroyed to so great an extent, the former did not appear to have suffered in any degree.'" 16.

As Dr. Webster observes, the preceding case is not calculated to render our notions on the functions of the respective columns of the cord much more clear or satisfactory than they were before. We must look to the further collection of facts for what light is to be thrown upon the subject. Our readers may probably remember that, in the volume of the Medico-Chirurgical Transactions published in 1840, and noticed in this Journal,* was a case reported by Mr. Stanley, in which the power of motion in the lower extremities was lost. In that case the posterior columns of the cord were diseased. The two cases taken together square indifferently with our received opinions.

### II. CASE OF BRONCHIAL CALCULUS, WITH OBSERVATIONS ON DISEASE OF THE BRONCHIAL GLANDS. By *John Charles Graham Tice*, M.D., Assistant Surgeon 8th (King's) Regt. of Foot.

Mr. J. A. aged 48, quarter-master, began to complain, Sept. 7, 1842, of pain in the right side, slightly affecting respiration. Pressure over the liver caused great uneasiness, the tongue was furred, the bowels were constipated, and there was a most disagreeable taste, in the mornings. Mercurial purgatives and bleeding were productive of little service, the pain of liver and dyspnœa increasing, with frequent paroxysms of cough. The fætor of breath augmented, and the horizontal posture was attended by a sense of suffocation. Bleeding, leeching, calomel, squills, antimony were

* January, 1841.

all employed. But the cough and difficulty of breathing grew aggravated, and the only position bringing any thing like ease, was leaning forwards, holding both knees.

Percussion and Auscultation elicited no disease of either heart or lungs. The patient now stated, that, "about three weeks prior to his illness he had partaken of a pear, and while part of it was passing down the œsophagus, he experienced a sense of soreness, and thought it remained in his chest.

"He pointed to a spot, a short distance below the clavicle, and a little to the left of the sternum. He added, that from that day up to the present, whatever he has swallowed, liquid or solid, appears to him not to pass beyond that spot.

"It was soon after the above event that he first noticed the fætor of his breath, which he represents, at times, as most intolerable."

In spite of all treatment, the cough became more frequent, and the paroxysms of longer duration, the dyspnœa, on assuming the recumbent posture being more alarming, with a sense of syncope at intervals. Dr. Wm. Stokes, who saw the patient, thought the disease was chiefly nervous and spasmodic irritation of the respiratory organs.

Soon after this, the breathing grew stridulous, the cough laryngeal, the horizontal position untenable. Between the thyroid and cricoid cartilages, the air passed as over a roughened surface. On the 11th Oct. laryngitis was suspected to have set in, and the symptoms increased in violence. After a distressing night, he was seized on the morning of the 18th October with a severe fit of coughing, which was at length subdued by help of an anodyne mixture; soon after taking which, he fell into a disturbed sleep. He became comatose after this, and, at last, after a deep inspiration he suddenly expired, six weeks having elapsed since the commencement.

*Autopsy.*—Right lung rather dark coloured and gorged with blood.

"On raising it and dividing its attachments, the knife suddenly entered a *cavity*, of the size of a pullet's egg, from which a most offensive odour was emitted. This cavity was formed by an abscess in a mass of enlarged bronchial glands, situated at the bifurcation of the trachea. It opened into the right bronchus, ulceration having destroyed a large portion of that tube. The inferior margin of the ulcerated opening was thickened and elevated: on the other side, the abscess communicated with the left bronchus, by an aperture of about half an inch in diameter, having inverted and thickened edges. Posteriorly, the abscess had made its way into the œsophagus by ulceration. The opening into that canal was capable of admitting a large-sized bougie. The abscess contained a quantity of calcareous matter, some very hard, some of a soft consistence. The whole, when first examined, was of a dark melanotic aspect. A triangular portion of the hard calculous matter had firmly wedged itself into the aperture communicating with the right bronchus.

"The mucous membrane in both tubes (above the ulceration) was marked by a deep blush, which extended nearly as high as the thyroid cartilage. A small ulcer was discovered towards the inner edge of the descending cornua of the thyroid cartilage on the right side." 25.

No other disease of any organ. Some of the calcareous mass, analyzed by Dr. Barton, was found to consist of phosphate of lime.

A careful perusal of the above case will be of service in tending to inform the reader of the nature of the symptoms, and what they indicated. The case is, itself, an instructive one.

III. ON CONGESTIVE PNEUMONIA, CONSEQUENT UPON SURGICAL OPERATIONS, DISEASES, AND INJURIES. By *John E. Erichsen*, Esq.

Mr. Erichsen's object is to direct attention to that form of Pneumonia, consequent on surgical operations and injuries, and *not* dependent upon the absorption of pus. He first points out the similarity of appearances between Pneumonia, in its first stage, and mere passive congestion of the lungs.

"The anatomical characters of the first stage of inflammation of the lungs, more especially when that disease assumes an asthenic type, are in all respects similar to those of mere passive congestion of those organs. The lungs, in both cases, being heavy from sanguineous engorgement, presenting externally a livid violet hue, mottled with spots of a dark red, or purple colour, and preserving the impression of the finger, as if œdematous. When pressed upon, they will be found to be more compact and solid than natural, scarcely crepitating. When cut into, a frothy, spumous, reddish fluid exudes in considerable quantity, and the pulmonary tissue will, at the same time, be found to be altered in its consistence, breaking down readily under even moderate pressure of the finger into a grumous pulpy mass. This friability of the tissue of the lung has been, by many, supposed only to occur in those congestions of that organ which were of an inflammatory nature, but it has been proved by the observations of Andral, and others, to be of no value as a diagnostic mark in distinguishing these from the mechanical engorgement that frequently supervenes but a few hours before death, and which, when it occurs, is probably the immediate cause of that fatal event." 31.

To avoid all source of fallacy, therefore, he considers as Pneumonia only those cases in which either one lung alone was affected, or some unequivocal evidence of inflammation existed, as hepatization or splenification of the pulmonary tissue, recent lymph or serum in the pleural sacs, or marked evidences of inflammation of the bronchial mucous membrane.

There is appended to the Paper, a Table of the Condition of the Lungs, in 62 cases of death, after surgical operations and injuries. These 62 cases arrange themselves under four classes :—those in which there were evident signs of Pneumonia, 28 in number ;—doubtful cases 11 in number ;—cases in which the lungs were found more or less diseased, but not inflamed or congested, 9 in number ;—cases in which the lungs were healthy, 14 in number.

"Thus it will be seen that on examining the lungs of sixty-two individuals who had died from various surgical diseases, operations and injuries, there were found marked evidences of Pneumonia in twenty-eight, or forty-five and one-tenth per cent. of the whole. Of the remainder it was doubtful in eleven whether the congestion that was met with was of an inflammatory or passive character. In nine others, the lungs were more or less diseased, being tuberculous in three cases, bronchitic in four, œdematous in one, and gorged with fluid blood in another : and in fourteen cases only, or little less than a quarter of the total amount—22·5 per cent.—were these organs healthy." 33.

In the 28 cases of Pneumonia, there was solidification in 17. In the remaining 11 cases, the Pneumonia was in the first stage. In three cases, the right lung only was affected—in three the left lung only—and, in twenty-two, both lungs ; though not to the same degree. In only two cases was the upper lobe found inflamed.

This form of Pneumonia is occasionally of a sthenic, more frequently of an asthenic character, like what is called the typhoid Pneumonia, where a passive congestion is superadded to the inflammatory state. "The blood, in this disease, stagnates in the lungs under the influence of certain causes, and a degree of irritation being at the same time set up, some inflammatory action is excited in the already congested part, which, however, is of a passive type, not being characterised by the formation of those secondary products that are the usual consequences of active, sthenic inflammation."

Among the causes disposing to Pneumonia, after injuries and operations, Mr. Erichsen gives first rank to the recumbent posture leading to congestion in the back parts of the lungs—and the debility, &c. produced by suppuration, hospital air, and so forth. Age, too, exerts an influence:—

| | |
|---|---|
| "The average age of the patients in whom inflammation of the lungs was found, was .. .. .. .. | 44·2 years |
| Of the doubtful cases .. .. .. .. | 39·4 — |
| Of the cases in which no inflammation or congestion of those organs occurred .. .. .. .. | 35·9 — |

So that the average difference in age between those cases in which Pneumonia occurred, and those in which it did not, amounted to 8·3 years." 38.

The recumbent posture, of itself, cannot go for very much. Irritation, fever, and suppuration, exert a considerable influence—long residence in a hospital the same. In the preceding cases, the average times that the patients lived, after admission into the hospital, were as follows:—

| | |
|---|---|
| "Cases in which the lungs were inflamed .. | 20·7 days. |
| Doubtful Cases .. .. .. .. .. | 12·3 — |
| Cases in which the lungs were found diseased, but not inflamed . .. .. .. | 22·7 — |
| Cases in which the lungs were healthy .. .. | 1.6 — |

"The reason of the high average of the time that those patients lived in whom the lungs were found diseased, but not inflamed, is, that of ten in that class, three died of phthisis on the thirtieth, fifty-fourth, and seventieth day respectively, after admission into the hospital. If these cases are excepted, we shall find that the remainder lived, on an average, only 8·4 days." 41.

After injuries, or operations, the surgeon should be on the watch for this affection. The stethoscope affords valuable aid, but from the circumstances of the case it is not always easy to apply it to the back of the chest, where the mischief takes place.

On treatment, Mr. Erichsen has little to say that is satisfactory. It is a more hopeful proceeding to attempt prevention than cure. Mr. E. suggests the changing, if possible, the recumbent position of the patient.

"The energies of the nervous system should be supported and increased by such stimuli and tonics as the patient may be able to bear; as, for instance, carbonate of ammonia, decoction of senega, quinine, and, in extreme cases of depression, wine and brandy. The inflammatory condition of the lungs should, at the same time, be combated by means of calomel, combined with minute doses of opium, and by counter-irritation in the form of dry-cupping, blistering, stimulating embrocations, or turpentine epithems." 44.

Bloodletting is out of the question.

As prophylactics, free ventilation—warm clothing—occasional changes, if possible, of position—the starched bandage, to enable patients to get about as soon as possible, are the means advised by Mr. Erichsen.

### IV. Researches into the Connexion existing between an Unnatural Degree of Compression of the Blood contained in the Renal Vessels, and the Presence of Certain Abnormal Matters in the Urine. By *George Robinson*, Esq.

Mr. Robinson has instituted an interesting series of experiments on the kidneys of rabbits, with the view of determining the physical results of compression of the blood.

One class of experiments consisted in applying a ligature on the renal vein, so as completely or partially to prevent the return of blood from it. This, of course, would lead to great congestion of the kidney, and to compression of the blood contained in it.

A second class of experiments consisted in the removal of one kidney, and ligature of the aorta below the other. By this means the arterial current would be directed with more than usual force on the remaining kidney.

For the experiments themselves we must refer to the Transactions, and content ourselves with a summary of Mr. Robinson's conclusions. He thus alludes to the two conditions by which the compression of the blood in its vessels is produced and regulated:—

"This compression is altogether dependent upon the co-existence and co-operation of two essential causes, each of which will, in different individuals, vary much in its amount of activity or degree of completeness.

"The momentum of the arterial blood arising from the contractions of the ventricle constitutes the active force from the operation of which the compression takes place. But as a counter resistance is required before an intense degree of the latter state can occur, it is only when some extraordinary obstruction to the free passage of the blood *through the smaller vessels* exists that the effects of an undue compression of that fluid are perceptible.

"It follows, therefore, that the momentum being equal in a number of cases, the intensity of the compression of the blood will be proportioned to the completeness of the obstruction; and, on the other hand, the impediment or obstruction being equally complete, the degree of compression will then be commensurate with the amount of the momentum.

"The whole of the preceding experiments, if carefully considered, will, I think, support this statement.

"They also prove—

"1. That simple compression of the blood in its smaller vessels will, in a direct ratio to the degree of intensity of that compression, cause the exudation of an albuminous fluid, of coagulating lymph, or the extravasation of blood. Its immediate effects, therefore, precisely resemble those of inflammation: and as it is well ascertained that both the essential causes of undue compression (viz. an obstruction or impediment to the flow of blood through the vessels of the inflamed part, and excessive action of the heart,) co-exist in that disease, it seems but reasonable to infer that the primary effects of inflammation, being identical with those of undue compression of the blood, are the mere consequences of that physical cause.

"2. That there is no relation between the composition of the effused matters and the extent of the dilatation of the coats of the vessels, as measured by the quantity of blood they contain." 64.

He presents a Table, corroborative of this position, and he repeats his belief, that the effusion of albumen and lymph through the coats of the vessels of the living body is dependent on and regulated by the degree of the compression of the blood contained within those vessels. In fact, he is a decided advocate for the application of ordinary physical laws to the explanation of the healthy and morbid phenomena of the living body.

### V. On Fatty Degeneration of the Arteries, with a Note on some other Fatty Degenerations. By *George Gulliver*, Esq. F.R.S.

Mr. Gulliver, whose contributions to microscopical and pathological knowledge are well known, has been carefully examining the deposits, atheromatous or steatomatous, between the internal and middle coats of arteries, and the opaque deposits on or in their internal membrane. The results of his investigations may be briefly summed up in the following manner:—

"1. The white or buff-coloured opaque spots of the inner membrane of the arteries are of a fatty nature.

"2. The soft matter, which has been generally called atheroma, and which often collects between the inner and middle coats, is also fatty.

"3. The fatty matter is frequently found in the substance of both these coats.

"4. A fatty degeneration of the tunics of the arteries is generally connected with that state of them which is the most frequent cause of aneurism, as well as of their obstruction, occlusion, or wasting, in aged people.

"5. The matter usually contains cholesterine and oleine, and often some margarine.

"6. The tunics of ossified arteries, as well as the bony plates, are often pervaded by the fatty substances just mentioned." 90.

These statements are substantially confirmed by the observations of Bizot, Cruveilhier, and Gluge, mentioned in a work on Pathological Anatomy, by Dr. Hasse, and referred to in a postscript by Mr. Gulliver.

Mr. G. is of opinion that other tissues are weakened and obstructed by the abnormal presence of fatty matter.

"In wasting of the testicle, and when the functions of that gland are impaired by lingering diseases or old age, the seminal tubes are often more or less obstructed by fatty matter, which occurs in free globules, and in more equal sized and minuter molecules, generally aggregated into comparatively large rounded or irregular masses, nearly opaque, and of a brown or dull yellowish colour." 93.

In every variety of consolidation of the lungs, more or less fatty matter will be found. In gangrene of the lungs, and in inflammation of the black lungs sometimes seen in old persons, fatty globules are generally rather numerous. The fatty condition of the liver, so familiar in phthisis, has usually been considered a consequence of impaired respiration. But the quantity of fatty matter in that organ has not seemed to Mr. Gulliver to be regularly increased in proportion to the diminution of the special function of the lungs. In children, cut off by various chronic diseases, but free from pulmonary consumption, the liver is not uncommonly surcharged with fatty matter.

### VI. Remarks on the Calculi in St. George's Hospital. By *Henry Bence Jones*, M.A. Cantab.

The Paper before us is the result of an analysis of a collection which now consists of 233 divided calculi, not including any duplicates, and 9 undivided.

Dr. Jones's first object is to shew that when the urates are deposited there is reason to suppose that little or no free acid can exist in the urine, and that consequently alkalies, however useful they may be in other respects, are not requisite in such cases to remove acidity.

His next object is to point out in what proportion of cases in this museum acid injections might have dissolved or partially removed the calculus.

" Of those calculi which have been divided,

46 are simple, that is, consisting throughout of one substance.

40 are compound, consisting throughout of a mixture of two or more substances.

147 are alternating.

Of these alternating calculi,

83 have a simple nucleus.

58 have a compound nucleus.

" If, instead of looking at the calculi from this point of view, we examine them for the purpose of seeing how often the same substance forms either a whole calculus, or occurs in a well-marked layer, we shall find that in this collection there are at least 450 distinct deposits. These I have arranged in the accompanying Table, from which it will be seen that

135 times uric acid occurs either alone or mixed with other substances.
222 — urate of ammonia, ditto.
163 — oxalate of lime, ditto.
139 — the phosphates, ditto.
80 — urate of ammonia with oxalate of lime.

The Table we must omit. Dr. Jones goes on to remark ;—

" In order that a deposit of urate of ammonia or of uric acid, with urate of ammonia, may take place, it is necessary that an excess of urate of ammonia, as compared with the quantity of water, should exist in the urine; yet this is not the case as regards a deposit of uric acid alone. It will be shown that for uric acid to be precipitated, no other unnatural state need be present except that of some free acid passing in excess out of the system. In this, perhaps, we may find a partial explanation of the frequent occurrence of uric acid calculi." 103.

Urate of ammonia appears to be increased in the urine from very slight causes. We cannot be surprised at its forming so frequent a constituent of calculi.

Oxalate of lime exists with uric acid, with urate of ammonia, and with the phosphates. It occurs 80 times in 450 with urate of ammonia, forming a distinct deposit. There is, indeed, a diathesis, in which urate of ammonia is formed at the same time with oxalate of lime. In the red deposit of rheumatism and of indigestion, he has found octohedral crystals of oxalate of lime, sometimes in large quantities.

Dr. Jones agrees with Dr. Prout, that urate of ammonia exists in the urine in the state of health. It appears that free acid and urate of ammonia cannot long exist in the same solution, and that whilst urate of ammonia only is deposited, no free acid can be present, although litmus paper may be reddened. It seems, therefore, most probable, that, when uric acid alone is deposited, much free acid must have been thrown out by the kidneys, and that thus all the urate of ammonia, which would otherwise have been present, must have been decomposed. To determine the frequency of this state of secretion, we must observe how often whole calculi consist of uric acid, *and* how often whole layers of this substance occur. This was 97 times in 450.

A small quantity of acid only partially decomposes the urate of ammonia in the urine; when, therefore, urate of ammonia is found mixed with uric acid, but little free acid is secreted by the kidneys Such a mixture was found to occur in 38 layers. Hence in 38 states out of 450, but little free acid was thrown off in the urine.

"When we find urate of ammonia alone, without any uric acid, forming a calculus or layer, we must consider that no free acid was removed by the kidneys, although the secretion may have been acid to test paper."

"The presence of the phosphates in a deposit generally implies a neutral or alkaline state of the urine. If such be the case, and the presence of uric acid implies an acid state from free acid, it would follow that uric acid and the phosphates must exist very rarely in the same deposit. When the calculus has consisted chiefly of the phosphates, I have not once found uric acid to exist with it. When traces of this acid have been present, careful examination showed it was in combination with some base. And when the calculus consisted chiefly of uric acid, the small ash which sometimes remains will rarely be found to consist of the phosphates.

"In the above Table the phosphates occur 139 times. Hence 139 times in 450 the urine must have been neutral or alkaline to test paper.

"If then phosphates indicate neutrality or alkalescence, and uric acid indicates free acid in the urine, we may conclude that the deposit of oxalate of lime, as it occurs in the above Table with uric acid, with urate of ammonia and with the phosphates, is independent of acidity and alkalescence, and that its presence in a layer does not indicate any particular state of the secretion.

"Now, as such layer implies a certain state of the urinary secretion, the 450 layers may be taken to represent 450 states of the urine.

"139 of these were neutral or alkaline, as so many times the phosphates are found to occur.

"311 were feebly or strongly acid to test paper.

"Of these 311, in 97 much free acid was passing from the system, as so often layers of uric acid occur.

38 but little free acid was thrown out, as so often mixed layers of urate of ammonia and uric acid appear in the Table.

117 no free acid passed, although litmus was reddened.

59 the state of the secretion is unknown, the oxalate of lime not offering any indication of it.

"Omitting these 59 oxalate of lime, there are then 117 states in which no free acid is passing from the system, and 135 in which little or much free acid was thrown out. From this it appears that in 252 cases of the uric acid diathesis, there were 117 in which no free acid was passing, and in these, alkalies would be of no benefit, so far as neutralising free acid in the urine is concerned: that is, in nearly every second case of the uric diathesis, there was but little if any free acid in the urine to be neutralised.

"In only 97 cases out of 252 was there much free acid secreted, or only twice in five cases were alkalies very necessary to remove the acidity of the urine; though in other cases these medicines might have been beneficial in some other respect." 107.

We arrive at the last subject for consideration—the cases in which acid injections might be serviceable.

"In 40 cases the phosphates or the phosphates and carbonates form the last layer.
"In 7 cases the whole calculus consisted of fusible deposit.
"In 5 cases the whole calculus consisted of phosphate of ammonia and magnesia.

"In these 52 cases out of 233, the calculus might have been lessened by the injection of dilute acids, and in 12 out of these the whole calculus might have been removed. This supposes however that in all these cases the calculi were in the bladder, and not in the kidneys, on which there is no satisfactory historical evidence.

"In addition to these 52 cases, there are 6 calculi which consist entirely of urate of ammonia and fusible deposit, and 19 in which fusible and urate of ammonia form the outside layer. In these cases, most probably, any acid injection would dissolve the fusible and decompose the urate of ammonia, and thus disintegrate the calculus. So that altogether in 75 out of 233 a solvent might have assisted in the removal, although in 18 only out of 233, or about 1 in 13, could the calculus have been entirely removed. For this Sir B. Brodie has shown dilute nitric acid sufficient. Perhaps at some future time lactic acid, which possesses a peculiar power of dissolving the phosphates, may be found even more rapidly efficacious." 109.

### VII. CASE OF ULCERATION OF THE INTERNAL JUGULAR VEIN, COMMUNICATING WITH AN ABSCESS. By *William Bloxam*, Esq.

The subject of this unusual occurrence was a child, five years of age, who had a glandular abscess in the neck, after scarlatina. Discharges of venous blood took place from it, and, in spite of the most careful compression, proved fatal. The internal jugular vein was found to communicate with the sac of the abscess by an oblong ulcerated aperture, five lines in its long diameter.

### VIII. SOME ACCOUNT OF AN HYSTERICAL AFFECTION OF THE VOCAL APPARATUS, WITH SEVERAL CASES. By *Oscar Clayton*, Esq.

The cases occurred in a charitable institution for female children, from the age of nine to that of fourteen. The cases were seventeen in number. They began with feverish and ended with hysterical characters. The main feature was spasmodic cough, which, in a few weeks, changed to sounds varying in the different patients; in some, resembling the double action of a large saw; in two, a shrill screaming expiration, following a quick catching inspiratory effort, much resembled the cry of a peacock; in another, the sound was such as is produced by blowing into a small metallic tube. In fact, it is difficult to conceive the dissonance and constancy of these sounds.

All kinds of remedies failing, the patients were sent home, when they recovered, but on coming together again, the same farce was re-enacted. An attempt was made to frighten the complaint away, by calling the girls together, and informing them that, if not better next morning, a hot iron would be applied upon their throats. Most of them ran away, but the complaint was not got rid of. A hot spatula on the throat did some good: time did more.

A good sample of the imitative disorders that sometimes prevail among communities of young persons, more especially females.

### IX. Case of Erectile Tumor in the Popliteal Space.—Removal. By *Robert Liston*, Esq., F.R.S.

Charles Reason, æt. 10, admitted into University College Hospital, Jan. 2, 1843, with a tumor in the right ham, of an oval shape, the long diameter being in the direction of the axis of the limb, situated in the upper part of the right popliteal space.

The swelling is about 3½ inches in length. The integuments covering it are not inflamed or discoloured, but perfectly natural. No pulsation is perceptible in it, nor can any '*bruit*' be detected by the ear. The pulsation of the artery can be felt in the popliteal space below the tumor, and those of the anterior tibial of the affected limb are natural. The tumor has a doughy, elastic feel, giving, when the limb is extended, a sensation much resembling the fluctuation that is produced by deeply-seated matter. When the limb is flexed, however, this sensation is less distinct, and the tumor has more the feel of an elastic solid mass, which is pretty moveable, and may be distinctly raised and separated from the bone.

The disease had first attracted attention eight years before. Various means, among others a seton, had been employed in vain. Mr. Liston supposing it a "tumor," possibly a fatty one, determined to cut it out.

An exploratory puncture was first made with a *knife*, which was half turned on its axis. There was venous bleeding to the extent of three ounces. Then another puncture was made elsewhere, and there was no bleeding.

"A free incision was made through the skin to the extent of about four inches. It was not adherent to the deeper parts. The fascia was now divided to an equal extent, and the surface of the tumor exposed. It had much the aspect of a fatty tumor, but its size was evidently much less than before the commencement of the operation. The opinion that the vein had been opened was now seen to be incorrect.

"The dissection was commenced on the outer side. The popliteal nerve was soon exposed, and the tumor, which was slightly adherent to it, carefully removed from it. After a troublesome dissection deep into the popliteal space, the tumor was found to be covered by muscle. The dissection was next proceeded with on the inner side, when it was soon found to be in like manner covered by muscular tissue, which was seen to be the semi-membranosus muscle embracing the tumor. During the manipulations necessary in this dissection the tumor has become much smaller than when first exposed on the division of the fascia. The substance of the semi-membranosus muscle was now cut into, and the morbid growth removed. The popliteal artery was not exposed in the course of the dissection. Only one vessel required ligature, and the patient was carried to bed, the wound being covered with lint dipped in cold water. A good deal of blood was necessarily lost during the operation, and the boy was somewhat faint." 124.

Bleeding occurred in the course of three hours, but was arrested by plugging the wound with lint. The wound ultimately did well.

"On making a section, the tumor was found to consist of a mass of most perfect erectile tissue as large as a hen's egg. This was completely surrounded by condensed cellular and fatty fatter. One part of the erectile tissue was more condensed than the rest, possibly where the seton had traversed it." 125.

Mr. Liston makes several remarks upon the case, the essence of which, however, consists in this—that an erectile tumor in the ham was mistaken for a fatty one. The circumstances were such as to render such an error excusable; they who have had anything to do with tumors, occupying deep situations, being too well aware of the uncertainty which attends their diagnosis. As in this instance, the history of the case sometimes rather misleads than assists.

We remember seeing one, in some respects, like this. The patient was a female of middle age, who had a tumor on the outside of the left thigh, about two-thirds down the limb. It looked like a fatty tumor, was considered to be one, and was operated on accordingly. It was found to be an erectile tumor in the substance of the vastus externus muscle. The tumor was not, as in this instance, circumscribed, and its extirpation was impossible. The patient died.

Mr. Liston mentions another interesting case, in which the sterno-mastoid muscle was involved.

### X. Two Cases of Osteosarcoma of the Thigh Bone, requiring Amputation of the Limb in both Instances. By *R. A. Frogley*, Esq., of Hounslow.

These cases are extremely interesting. There is a great similarity between them. Their titles explain their nature, and it is only requisite to add, that the necessary operations appear to have been performed with equal ability and success by Mr. Frogley.

### XI. Remarks on Cancrum Oris, and the Gangrenous Erosion of the Cheek of Mr. Dease and Dr. Underwood, and more particularly on the Efficacy of the Chlorate of Potash, in the Treatment of those Diseases. By *Henry Hunt*, M.D.

The main point of the Paper is to recommend the use of the chlorate of potass. This may be preceded, or not, by an aperient, according to circumstances.

"The quantity of the salt that I have been in the habit of prescribing varies from twenty to sixty grains, according to the age of the child, in divided doses in twenty-four hours, dissolved in water; the beneficial effect is often observed on the following day, almost always on the second; the disagreeable fætor soon lessens, the sores put on a healthy reparative action, the dribbling of saliva diminishes, and if there is mere ulceration it very speedily heals, if there is an eschar, it soon separates, and the sore granulates kindly. In no other disease did I ever see the beneficial effects of any medicine so soon manifested, as that of the chlorate of potash in these diseases. It is sometimes advisable, indeed necessary, that the aperient should be occasionally repeated."

Four cases are related—one by Mr. Cæsar Hawkins. They speak very favourably for the remedy, which is one that deserves a trial.

### XII. Case of Ulceration of the Pulmonary Artery into an Abscess of the Lungs. With Remarks by *John Dalrymple*, Esq. By *William Crowfoot*, Jun. Esq. Beccles.

The following case, taken in connexion with that of ulceration of the jugular vein, transcribed a page or two back, is not uninteresting.

Mr. L. B. aged 36, of a consumptive family, had had symptoms of phthisis, more or less, from the Autumn of 1841 to November, 1842, when he was attacked with active hæmoptysis. The blood was always expectorated without effort. The physical signs were these—the ribs on the left side were contracted and flattened when compared with those of the right side, the upper part of the left side of the chest was dull upon percussion, and the respiratory murmur was absent in that situation; the right ride presented no abnormal sound. At the end of a month he sank.

*Examination.*—Adhesions between the pleura pulmonalis and costalis of the left side—pleura pulmonalis of both sides, presenting the spotted appearance from strumous deposit. "The upper part of the left lung was entirely occupied by a large cavity containing about half a pound of grumous and coagulated blood; the walls of the cavity were composed of the parenchymatous structure of the lungs, condensed and solidified by pressure. Upon careful examination we found the pulmonary artery opening into the cavity at the distance of two inches from its bifurcation by an aperture as large as a crowquill."

### XIII. Cases of Strangulated Hernia, reduced, "*en Masse*." With Observations. By *James Luke*, Esq. Surgeon to the London and St. Luke's Hospitals, and Lecturer on Surgery.

The occurrence which forms the subject of Mr. Luke's Paper is the reduction of the hernial protrusion, sac and all—an occurrence esteemed, perhaps, rarer than it actually is.

It would seem that five cases of reduction *en masse* have come under Mr. Luke's notice. A great similarity of circumstances appears to have attended three of them. "In each case, the hernia was oblique inguinal—and reduced by the patient's own efforts. In each the precise nature of the case was not known during life, and no operative attempt was made to afford relief; and in each the *post mortem* examination discovered a hernial tumor in the neighbourhood of the internal ring, reduced through the abdominal parietes, but lying exteriorly to their general peritoneal investment. On opening the tumor, in each its fundus lay below the level of the ring, towards the cavity of the pelvis, the contents being found in a state of sphacelus, and strictured by the neck of the sac, in which they were enclosed."

Mr. Luke relates the particulars of two cases, for which we must refer to his Paper, and proceed to the conclusions that he draws. The cases themselves, however, are extremely interesting.

Mr. Luke observes that the practice adopted by all prudent practitioners in doubtful cases of intestinal obstruction, of examining the abdominal apertures for the existence of hernial protrusion, may deceive, unless combined with inquiries into the previous history of the case. It is not enough that no tumor *exists*, for it must also be ascertained whether any such tumor has previously *existed*.

When a hernia has occurred, the sac generally soon acquires adhesions to the neighbouring parts, which tends to obviate the risk of its return with its contents under the operation of the taxis. Yet, in all the cases related, the hernia had been of some years' continuance, but was reduced without the employment of much force notwithstanding.

"The presence of sac, even without hernial contents, causes an abnormal fullness in the part, easily ascertainable by examination. The absence of such fullness in a part, when hernia is known to have previously descended, necessarily leads to the conclusion, that the sac upon which it depended has been displaced, and probably returned, together with the hernia.

"The sac in inguinal hernia, below the external ring, becomes united with the spermatic chord, whereby the latter is usually rendered indistinct and obscure. The absence of that indistinctness and obscurity implies the removal of the cause which previously produced them, and therefore that the sac has been displaced. The continuance of the indistinctness and obscurity leads to a directly contrary conclusion.

"When a hernia descends from the abdomen, the aperture through which it descends is always enlarged and dilated. This fact is ascertainable by the introduction of a finger, a circumstance which becomes available to the diagnosis in these cases.

"Should a large aperture be detected, a previous hernial descent may be inferred.

"Under ordinary circumstances of hernia, when the contents are reduced into the abdomen, the area of the aperture is occupied by the remaining sac, while its margins are rendered more or less obscure. If, then, a large aperture be found free and unobstructed, with its margins unobscured, there is raised not only a presumptive evidence of the previous protrusion of a hernia at the part, but also the further evidence of the displacement and probable return into the abdomen of the sac by which the hernia had been invested.

"We are led to a contrary conclusion by contrary circumstances." 176.

If a tumor be discoverable in the inguinal canal, of course, the diagnosis is comparatively clear. Supposing none such, still, if reduction *en masse* has been effected, the inflammation of the hernial contents *may* cause a circumscribed pain in the seat which it occupies, while a fullness, or even the rounded form of the hernia deeply situated within the abdominal parietes, may possibly be cognizable upon a minute examination.

Now, if circumstances justify the suspicion of reduction *en masse* of the tumor, they will also justify attempts to cause its reprotrusion. So the patient may be placed erect, and directed to cough forcibly, strain, and make exertion.

This seems likely to be of use, when the hernial tumor is in the inguinal canal, or at the inner ring—if it be within the abdomen, it is less to be depended on.

Mr. Luke argues in favour of an operation of exploration, and dwells on a close observation of the circumstances disclosed by the incisions.

"By the perfect exposure of the external inguinal ring, which the cutaneous incision affords, the size of the aperture, together with the extent to which it is occupied by structures passing through it, are clearly made manifest, and the same inferences drawn from the observance of these particulars, as suggested in the mere manual examination. For if the size of the ring be normal, a hernia has not descended through it; or, if it be larger than the normal state, yet occupied by an empty sac, an evidence of the previous existence of a hernia, together with an evidence of the reduction of the hernia without the sac being also reduced, is established. But should the ring be found large, and free from other structures than the chord, and if the chord be distinct and unobscured by the presence of sac, and a void is found where fullness is to be expected from the previous history of the case, a strong presumptive evidence on the contrary side is established, that the hernia, together with its investing sac, is reduced." 181.

The inguinal canal is next laid open.

"It will be recollected that the ordinary oblique inguinal hernia, during its passage through the canal, lies anterior to the spermatic chord. The hernial sac, when left empty after the reduction of its contents, occupies the same relative situation, and consequently overlays and obscures the chord after the canal is laid open. If the reverse of this is found in a case where a hernial descent is known to have previously existed, and the chord is ascertained to be clearly and distinctly brought into view throughout the whole extent of the canal, we may justly conclude that the distinctness and clearness with which the chord is seen are caused by the removal and consequent reduction of the hernial sac from over it, which reduction can be effected in no other direction than into the abdomen.

"Again, it is well known to all operators on strangulated hernia, that there is usually found a condensed cellular capsule immediately investing the sac, which, in the performance of an operation, assumes a laminated appearance, and often passes for layers of fascia. This cellular capsule has but little connection with the sac, and will remain even when the sac has been reduced. It will, therefore, be worth while to seek for such capsule in our explorations; for, if found, and ascertained to be empty, the circumstance is of a very conclusive character, and moreover will afford a direct clue to the situation of the hernia.

"A finger, introduced through an opening made in such capsule, will be conducted towards or through the internal ring, beyond which it will be brought into contact with the hernial tumor itself, having in the introduction passed through the same channel by which the reduction was effected. It must not be expected that such capsule will be found in all cases, because it might escape notice by reason of its tenuity, or, in reductions of some duration before the performance of operation, adhesions and obliteration may be caused by inflammation; yet when found, it is a most valuable adjunct to the other means of diagnosis.

"The indications to be noticed at the internal are of a similar nature to those mentioned as being found at the external ring, and relate to the size of the aperture and the structures by which it is occupied. With reference to its size, it may generally be expected to be abnormally large, because before proceeding to perform an exploring operation, there will probably be some account received of a hernial descent having occurred, which descent necessarily implies that it has passed below this ring, and consequently through it. For that reason the ring may be expected to be large, and its borders defined; while its area will or will not be occupied by hernial sac, and the same conclusions drawn from the particular ascertained, as from the same occurrence at the external ring." 183.

Hitherto, there has been no damage done to the peritoneum. But, so far, the circumstances have merely afforded a presumption. Mr. Luke advises the introduction of a finger through the internal ring, and passing it from side to side.

"Should a hernial tumor be present, it will at once be recognised, and found lying externally to the general peritoneal membrane, although within the parietes, and presenting a rounded surface and tense feel. Should a tumor be not present, the circumstance may be ascertained by observing the smooth surface of the peritoneum, and the continued adhesions which it maintains with the parietes immediately surrounding the ring.

"If doubt still exists, an enlargement of the internal ring, by division of the adjoining transversalis fascia, will afford a clearer exposition of parts, and a more decisive evidence for either an affirmative or a negative conclusion; and thus an exploration may be conducted to its termination, without the necessity of any peritoneal section.

"When the doubts have been resolved in the affirmative, by the discovery of a hernial tumor, the tumor may be brought into the inguinal canal, so as to occupy its former situation before reduction, by enlarging the ring to the requisite extent for its passage. It may afterwards be opened, and its contents dealt with according to their condition, as under the ordinary circumstances of common operations." 184.

It must not be forgotten that, in cases of reduction *en masse*, the stricture is in the neck of the sac. This should be opened, and the neck freely divided. "It should be recollected, also, that the adhesion of the sac to surrounding parts has been severed, and that, consequently, the sac will be liable to be again reduced, during the reduction of the contents into the abdomen, unless caution be used for its prevention. The danger of this occurrence may be always obviated by the introduction of the finger through the neck of the sac, after the contents have been reduced; for thus the fact of their perfect liberation may be readily ascertained."

It may happen, though rarely, that the contents of a hernia are reduced into the abdomen, with the strangulation continued on them by other causes than the neck of the sack.

"Thus the hernial contents may be so situated, that some portions may become strangulated by others, or by adhesions formed during the progress of inflammation, or by apertures of the omentum or mesentery admitting through them portions of intestine. These strangulations may take place either entirely within the abdomen, or within a hernial sac protruded from it. If in the latter situation, and the ordinary symptoms of intestinal obstruction supervene, the taxis may be applied in the usual way, and apparently at first with success, because neither the ring nor the neck of the sac, in such cases, may offer any obstacle of sufficient magnitude to prevent the passage of the tumor through the parietes. Yet, as the strangulation is caused by the involvement of the contained parts with each other, and wholly independent of the apertures through which they passed from the abdomen, their reduction is not followed by the expected relief. A certain similarity to the cases related is thus caused, and the suspicion of a reduction *en masse* is thus created.

"The suspicion may lead to an operation of exploration of the part. But the signs of a reduction *en masse* before mentioned, as coming under the cognizance of the surgeon during the progress of the operation, will be found wanting; the empty sac, with the obscurity of the chord and rings consequent upon its continued presence, becoming so many evidences against such reduction. Neither are there any local signs from which an unequivocal opinion can be formed.

"It is probable, that if the parts strangulated remain in the neighbourhood of the ring, a local and obscurely felt tumor, painful over a circumscribed space, may be discovered upon examination, especially after opening the inguinal canal. Thus may be afforded some grounds for suspecting the true nature of the case, yet not sufficiently decisive to render a section of the peritoneum anything more or less than a mere speculative proceeding, with all the contingencies of its doing good or harm to the patient's prospects of recovery." 187.

A valuable paper. The rest of the Volume in our next.

